# Hepatic sarcoidosis with symptomatic portal hypertension: A report of 12 cases with review of the literature

**DOI:** 10.3389/fmed.2022.995042

**Published:** 2022-12-22

**Authors:** Maxime Fauter, Geoffrey Rossi, Ayoub Drissi-Bakhkhat, Marianne Latournerie, Mathieu Gerfaud-Valentin, Isabelle Durieu, Yvan Jamilloux, François Bailly, Matthieu Mahevas, Pascal Sève

**Affiliations:** ^1^Department of Internal Medicine, Croix-Rousse Hospital, Lyon, France; ^2^Department of Internal Medicine, Henri-Mondor Hospital, Assistance Publique – Hôpitaux de Paris, Université Paris-Est Créteil, Créteil, France; ^3^Department of Hepato-Gastroenterology, Centre Hospitalier Universitaire Dijon Bourgogne, Dijon, France; ^4^Department of Internal Medicine, Centre Hospitalier Universitaire Lyon Sud, Pierre-Bénite, France; ^5^RESHAPE, INSERM U1290, Lyon University, University Claude-Bernard Lyon 1, Lyon, France; ^6^INSERM U1111, Centre International de Recherche en Infectiologie/International Research Center in Infectiology (CIRI), University Claude-Bernard Lyon 1, Villeurbanne, France; ^7^Department of Hepato-Gastroenterology, Croix-Rousse Hospital, Lyon, France; ^8^Pôle IMER, Hospices Civils de Lyon, Lyon, France

**Keywords:** hepatic sarcoidosis, portal hypertension, ascites, variceal bleeding, cirrhosis

## Abstract

**Introduction:**

Sarcoidosis is a systemic granulomatosis of unknown etiology, characterized by the presence of immune granulomas. Liver damage is a relatively common extra-pulmonary manifestation, occurring in 3.6–30% of cases. Some patients can develop symptomatic portal hypertension (PH). Few series have evaluated the prognosis of symptomatic PH as well as the efficacy and safety of specific treatment on this complication.

**Methods:**

This is a multicenter retrospective study of cases of histologically proven hepatic sarcoidosis with symptomatic PH (ascites, digestive hemorrhage) and/or hepatic encephalopathy. Demographic characteristics, comorbidities, clinical manifestations of sarcoidosis, biological data, imaging study of the liver, treatment, and clinical outcomes were collected.

**Results:**

Twelve patients were identified, with a mean follow-up of 140 months. The M/F ratio was 1 and Caucasian origin was the most represented (75%). Seven patients presented with hepatic comorbidities: metabolic syndrome, chronic alcoholism or chronic viral hepatitis. Apart from hepatic involvement, mediastino-pulmonary involvement was the most common followed by osteoarticular and skin. Liver damage was inaugural in two thirds of cases. Nine patients developed ascites, six presented esophageal varices complicated by gastrointestinal bleeding. Three patients presented with both ascites and variceal bleeding. One case of hepatic encephalopathy was observed. Five patients presented signs of hepatocellular insufficiency during follow-up, of whom three had hepatic comorbidities. Eight out of 12 patients required second-line treatment after failure of corticosteroids, three patients underwent ligation of esophageal varices but with recurrent digestive bleeding in all cases. Two patients benefited from a transjugular intrahepatic portosystemic shunt (TIPS), also with poor result. At the end of follow-up, five patients were alive and seven patients died. Two patients received a liver transplant, with good result and without recurrence of sarcoidosis on the transplant thereafter. Two patients had quiet sarcoidosis on low dose of corticosteroids and one patient was lost to follow-up.

**Conclusion:**

Symptomatic PH related to hepatic sarcoidosis is a severe complication, with high morbidity and mortality, and frequent failure of specific treatments of PH. Early management of these patients, with detection of hepatic comorbidities seems important. In case of therapeutic failure, liver transplantation is an option to consider.

## Introduction

Sarcoidosis is a systemic inflammatory disorder of unknown etiology characterized by non-caseating granulomas, most commonly involving lungs and hilar lymph nodes (1). The prevalence of sarcoidosis worldwide is difficult to determine, with higher incidence of the disease in northern (about 60 per 100,000) than in southern European countries (2). Liver involvement is a relatively common extra-pulmonary manifestation, occurring clinically significant in 3.6–30% of cases (3–6), whereas autopsy series have shown histological hepatic involvement in up to 50–70% of patients. African American patients seem to have more frequent liver involvement (4). Hepatic sarcoidosis is therefore mainly asymptomatic even in the setting of abnormal liver function tests, but can also manifest with non-specific symptoms, such as fever, abdominal pain, pruritus or fatigue (1).

Concerning laboratory abnormalities, most patients have mild biochemical abnormalities including an elevated alkaline phosphatase (ALP) and gamma glutamyl transpeptidase (GGT), elevation in alanine aminotransferase (ALT) and aspartate aminotransferase (AST) levels being less common, suggesting the infiltrative nature of sarcoid granulomas with cholestatic liver injury and less hepatocellular destruction (7, 8). Moreover, the severity of liver-test abnormalities has been shown to be significantly associated with extensiveness of granulomatous inflammation (8).

Even if hepatic sarcoidosis is rarely severe, some patients can develop serious complications such as symptomatic portal hypertension (PH) (2). Common complications of PH are ascites, variceal bleeding or portal vein thrombosis. PH in hepatic sarcoidosis (9) can result from cirrhosis or non-cirrhotic mechanisms, by presinusoidal block due to portal granulomas, or portal or vein hepatic thrombosis (5, 10, 11). It may be inaugural or complicate hepatic sarcoidosis history (12). Given the chronic nature of the disease, it is estimated that 3–28.6% of patients will progress to cirrhosis, as a result of parenchymal fibrosis secondary to granulomatous inflammation or vascular injury (10, 13). Eventually, progression of the disease can lead toward liver failure requiring liver transplantation or death (14).

Corticosteroids and ursodeoxycholic acid are considered as the mainstay of therapy, although refractory cases may require other immunosuppressive agents such as azathioprine or methotrexate (1). However, there are no randomized controlled trials evaluating hepatic sarcoidosis treatments and questions remain regarding what to treat, with what and when (12).

In view of the rarity of serious hepatic sarcoidosis cases, mainly due to complications of PH, there are only few descriptions of the therapeutic approach and prognosis of these patients, mostly in the form of case reports (13).

In this present study, we describe the clinical features and prognosis of 12 patients with histologically proven hepatic sarcoidosis, complicated by symptomatic PH, as well as the tolerance and efficacy of the treatments used. Additionally, we review the relevant hepatic sarcoidosis literature.

## Materials and methods

In this retrospective multicentric study, we aimed to identify patients with severe hepatic sarcoidosis, seen between January 1st, 2000 and December 31, 2020. After having highlighted several observations in our center, we made a national call for observations *via* the “Groupe Sarcoïdose Francophone” to retrieve more. We included patients aged ≥ 18 years, with a proven granulomatous hepatitis attributed to sarcoidosis, according to ATS/ERJ/WASOG criteria (15), and symptomatic PH (ascites or gastrointestinal bleeding) or hepatic encephalopathy, at diagnosis or during follow-up.

Differential diagnoses of granulomatous diseases, autoimmune disorders, especially primary biliary cholangitis, malignancies or infectious diseases, especially mycobacteria, were excluded, including autoantibody testing, infectious serology and/or microbiological molecular diagnosis on liver biopsy (16).

Demographic characteristics, comorbidities, clinical manifestations, extrahepatic involvement of sarcoidosis, biological data, imaging study of the liver, treatment, and clinical outcomes were collected. Liver-test abnormalities were taken into account if one of the following liver tests exceeded the level of the upper limit of normal (ULN) 1.5 times: ALP, GGT, ALT, and AST. Serum angiotensin converting enzyme (ACE) was considered elevated if its level exceeded 1.5 times normal. Lung involvement was based on Scadding’s radiological stages of sarcoidosis also known as stage I: bilateral hilar lymphadenopathy, stage II: bilateral hilar lymphadenopathy with pulmonary infiltrate, stage III: pulmonary infiltrate alone, stage IV: fibrosis (17). Cirrhosis was defined by its pathological features on microscopy: the presence of regenerating nodules of hepatocytes and the presence of fibrosis.

The efficacy and tolerance of the treatments were also studied. Complete response was defined as normalization of the liver tests, partial response as >50% improvement, and failure as use of other therapy, <50% improvement, or no improvement in liver tests (5). All treatment side effects were noted.

### Literature review

We searched Medline for original articles published that included an abstract in English or French from January 1988 to December 2021, using the following key words: “hepatic sarcoidosis,” “PH,” “ascites,” “variceal bleeding,” “hepatic encephalopathy,” as well as specific search terms relevant to each. Other spelling and abbreviation were included in the search.

### Statistics analysis and ethics

Descriptive statistics were used to summarize the cohort demographics. Follow-up was continued until death, migration out of system or December 31, 2020. This study was approved by the Institutional Review Boards (N°20-116) and was registered on www.clinicaltrials.gov (NCT04459897).

## Results

### Clinical characteristics

Twelve patients with histologically proven hepatic sarcoidosis and symptomatic PH were identified. All patients came from academic centers in France. First, we identified 51 patients with proven hepatic sarcoidosis seen at our institution, among which 9 patients (17.6%) had PH and 7 (13.7%) met inclusion criteria. We then looked in a cohort of 11 patients from the Assistance Publique—Hopitaux de Paris, in which 4 had symptomatic PH. Finally, we included one more observation from the academic center of Dijon. The description of the 12 patients is summarized in Table 1.

**TABLE 1:** Clinical and biological characteristics of the 12 patients with symptomatic portal hypertension.

Patient	Age	Sex	Ethnic group	Hepatic comorbidities	Hepatic symptoms	Non-specific symptoms	LFT at diagnosis	Elevated ACE	Cirrhosis at diagnosis	Extrahepatic disease
1	48	F	Caucasian	Obesity	Ascites, variceal bleeding	Asthenia, weight loss	Cholestasis	Yes	Yes	MP, skin
2	47	M	Caucasian	Obesity alcohol	Ascites		Cytolysis	Yes	Yes	MP, kidney
3	76	M	Caucasian	Alcohol	Variceal bleeding		Cholestasis	No	Yes	MP, skin, joint, kidney
4	29	F	African	Chronic HCV	Ascites		Cytolysis	No	No	MP
5	30	M	Caucasian		Variceal bleeding	Abdominal pain	Normal	No	No	MP
6	43	F	Afro-Caribbean	Obesity	Ascites	Asthenia, weight loss	Cytolysis cholestasis	No	No	MP, eye, skin, heart, joint
7	40	F	Caucasian	Obesity metabolic syndrome	Ascites		Cholestasis	Yes	No	MP
8	48	M	African	Chronic HBV	Ascites		Cytolysis	Yes	No	None
9	45	F	Caucasian	Metabolic syndrome	Ascites variceal bleeding	Asthenia, weight loss	Cytolysis cholestasis	No	No	MP
10	62	F	Caucasian	Obesity metabolic syndrome	Variceal bleeding encephalopathy	Abdominal pain	Cytolysis cholestasis	Yes	Yes	MP
11	57	M	Caucasian		Ascites variceal bleeding		Cholestasis	No	Yes	CNS, kidney
12	68	M	Caucasian		Ascites	Asthenia, weight loss	Cholestasis	No	No	None

F, female; M, male; HCV, hepatitis C virus; HBV, hepatitis B virus; MP, mediastino-pulmonary; CNS, central nervous system.

The mean age at diagnosis of sarcoidosis was 49.4 years (range, 29–76). The mean duration of follow-up was 140 months (range, 12–425). The male/female ratio was 1. Nine patients were of Caucasian origin (75%), two were African and one Afro-Caribbean. Seven patients had hepatic comorbidities: metabolic syndrome (three patients), chronic alcoholism or chronic viral hepatitis (two patients each). Four patients were overweight (IMC ≥ 25) and five were obese (IMC ≥ 30). One patient had a history of treated tuberculosis (with negative mycobacterium PCR on liver biopsy). Three patients had positive anti-nuclear antibodies, at a low level and without specificity.

Apart from hepatic involvement, mediastino-pulmonary involvement was the most common (9/12) followed by skin (3/12) and osteoarticular (2/12) involvement. Cardiac, renal, ophthalmologic or nervous system involvements were rarer (present in one patient each).

Liver damage was inaugural in two thirds of cases. Few patients presented non-specific clinical symptoms such as weight loss or asthenia in four patients (33.3%), or abdominal pain in two patients (16.6%). No patient had fever.

At diagnosis of hepatic sarcoidosis, liver test abnormalities were observed in 11/12 patients (91.6%) with cholestasis in five patients, cytolysis in three patients and combined abnormalities in three patients. Five patients (41.6%) had elevated ACE, four patients (33.3%) had hypercalcemia, and eight (66.6%) had polyclonal hypergammaglobulinemia.

A morphological abnormality (hepatomegaly or hepatic nodules) was present in nine patients (75%). The chest X-ray was normal in six patients (50%), at stage I in three patients (25%), stage II in two (16.6%) and stage IV in one patient (8.3%).

After a median follow-up of 90 months (12–425 months), a total of eight patients developed liver cirrhosis. Cirrhosis was found at diagnosis of hepatic sarcoidosis in six patients, while the other two patients developed cirrhosis during follow-up. There were four cases of PH without cirrhosis. Nine patients (75%) developed ascites, eight (66.6%) presented esophageal varices, six of which (50%) were complicated by gastrointestinal bleeding. For these patients, signs of PH were present in four patients at diagnosis of hepatic sarcoidosis (three with esophageal varices and two with ascites). Three patients presented with both ascites and variceal bleeding. Six patients had splenomegaly. Only one case of hepatic encephalopathy was observed, concomitant with variceal bleeding. Five patients presented biological stigmas of hepatocellular failure (prothrombin rate < 50% with reduced factor V, bilirubin > 35μmol/L) during follow-up, of whom three had hepatic comorbidities. No case of hepatocellular carcinoma was observed, as well as no Budd-Chiari syndrome or portal vein thrombosis.

### Efficacy and tolerance of treatment

Concerning therapeutic management, 10 out of 12 patients (83.3%) received oral corticosteroid therapy, between 0.3 and 1 mg/kg, among which eight (66.6%) required second-line treatment, notably azathioprine in five of them and mycophenolate mofetil in two patients. Hydroxychloroquine and methotrexate were also used in two patients each. There was no use of anti-TNF-type biotherapy. Ursodeoxycholic acid was used for cholestatic disease in eight patients (66.6%). Corticosteroids allowed a complete response in one patient (8%) with mixed cirrhosis, a partial response in another patient, and failure in the others, with no improvement of liver test abnormalities or need for a second treatment. Azathioprine was used each time as a second-line treatment, in the face of worsening on corticosteroids. Mycophenolate mofetil was introduced for extra-hepatic involvement (renal sarcoidosis and post liver transplant therapy). In this regard, the decision to start treatment (corticosteroids or immunosuppressive agents) was also guided by non-hepatic sarcoidosis in five of 12 patients. Hydroxychloroquine was used in one patient, in association with corticosteroids, for hypercalcemia, and in another patient for cortisone sparing purposes. Methotrexate was introduced as a second-line treatment in two patients, for skin and articular involvement for one, and again for cortisone sparing purposes for the other. Among the six patients who required second-line treatment, partial response was observed in two patients (after third-line therapy) and failure in the four others, knowing that 3 patients required more than one treatment each. All but one of the patients who required second-line therapy died during follow-up, except for the transplant patients.

The main adverse events for corticosteroids were cortico-induced diabetes in three patients, and severe acne in one patient. There was no worsening of liver function on azathioprine. Five patients presented infectious complications requiring hospitalization, and resulting in death in two patients. Both were treated by immunosuppressive drugs (azathioprine or mycophenolate mofetil) and had advanced cirrhosis (Child-Pugh Score ≥ C10), the first one due to fungemia and the second due to an infection of the ascites fluid with digestive hemorrhage.

For PH management, three patients underwent ligation of esophageal varices but with recurrence of digestive bleeding in all cases. Two patients also benefited from a transjugular intrahepatic portosystemic shunt (TIPS) with nonetheless a poor result (postoperative death due to digestive hemorrhage for one patient and recurrence of ascites for the other). Summary of the therapeutic management of the 12 patients is detailed in Table 2.

**TABLE 2:** Therapeutic management of the 12 patients with symptomatic portal hypertension.

Patient	1st line	Response	2nd line	Response	3rd line	Response	Specific PH management	Follow-up (months)	Evolution
1	Steroids	Failure	MTX	Failure	AZT	Partial		18	Lost to follow-up ascites and EV
2	None mixed cirrhosis				Liver transplantation	Complete		20	Alive no symptom
3	Steroids	Complete						59	Alive no symptom
4	Steroids, HCQ	Failure	MTX	Failure	AZT	Partial	Sclerotherapy	398	Death GI bleeding
5	Steroids	Failure						425	Death infection
6	Steroids	Failure	AZT	Failure	Liver transplantation	Complete		252	Alive no symptom
7	Steroids	Partial						84	Alive no symptom
8	Steroids	Failure					TIPS	156	Death infection
9	Steroids	Failure	AZT	Failure			Sclerotherapy	144	Death GI bleeding
10	None mixed cirrhosis							96	Death GI bleeding
11	Steroids	Failure	MMF	Failure			Sclerotherapy TIPS	12	Death GI bleeding
12	Steroids, HCQ	Failure	AZT	Failure				26	Death hypercalcemia

MTX, methotrexate; AZT, azathioprine, HCQ, hydroxychloroquine; MMF, mycophenolate mofetil; TIPS, transjugular intrahepatic portosystemic shunt; GI bleeding, gastrointestinal bleeding.

### Outcome

At the end of the follow-up, seven patients (58.3%) died (four patients in a context of gastrointestinal bleeding, two after infectious complications and one patient after cardiopulmonary arrest of unknown etiology, in a context of hypercalcemia). Five patients were alive, among which two were responders to corticosteroids, with quiet sarcoidosis without current complications of PH, on low dose of corticosteroids (≤5 mg/day), and one patient was lost to follow-up, while he presented ascites and esophageal varices, treated by azathioprine. Finally, two patients received a liver transplant, the first in a context of mixed cirrhosis (ethylic and sarcoidosis origin) and the second for refractory ascites after a long course of sarcoidosis, with a good result, without recurrence of the sarcoidosis thereafter.

## Discussion

In the present study, we report a series of 12 patients with histologically proven hepatic sarcoidosis and symptomatic PH, reflected by ascites, digestive hemorrhages and more rarely by hepatic encephalopathy. We found low efficacy and rather poor tolerance of the treatments used, whether for sarcoidosis or PH management. At the end of the follow-up, a significant proportion of patients died, including four patients in a context of gastrointestinal bleeding, suggesting the clear impact of PH on mortality of these patients. Finally, liver transplantation was performed in two patients, with a good outcome, and without recurrence of sarcoidosis afterward.

There are no formalized diagnostic criteria for hepatic sarcoidosis. Giving that there are no specific symptoms, diagnosis involves typically a clinical history of systemic sarcoidosis and a liver biopsy evidence of non-caseating granulomas (12). The prevalence of clinically significant hepatic involvement in patients with systemic sarcoidosis varies widely between studies, ranging from 3.6 to 30%, while asymptomatic disease can be present at a greater incidence rate (1, 5, 13). Despite its small size, the main strength of this study is that it is based solely on histologically proven hepatic sarcoidosis, whereas most other studies include sarcoidosis with probable but unproven liver involvement.

Otherwise, clinical manifestations of sarcoidosis are known to vary among ethnic groups. Despite report of an association between African American race and severity of liver involvement (18), we did not find a significant impact of race on this cohort, probably because of the non-American population. Our study is limited by a small sample size, and almost all the patients were Caucasians.

A recent retrospective study showed that the most common symptoms of hepatic sarcoidosis were fatigue, pruritus, weight loss, and hepatomegaly (12). In this study, one third of the patients showed non-specific clinical symptoms (weight loss, asthenia, abdominal pain).

Another clinical consequence of hepatic sarcoidosis can be the occurrence of PH, whose incidence is estimated between 10 and 50% according to studies (5, 9, 10, 13, 19). In our center cohort, 17.6% of patients with hepatic sarcoidosis developed PH, among which 77.8% had symptomatic PH. It may be revealed by ascites or gastrointestinal bleeding from esophageal varices, or discovered incidentally through imagery. However, symptomatic PH revealing hepatic sarcoidosis seems rare, with only 2/12 patients (with ascites) in our cohort, and < 10% of the patients in the literature (5, 12). Variceal bleeding at diagnosis is exceptional and limited to case reports (20). Prevalence of symptomatic PH is not clear, as the few studies about hepatic sarcoidosis only report the presence of PH, without specifying its consequences, and the rare symptomatic patients are limited to case reports or small retrospective studies (5, 11, 12, 20–26). While digestive bleeding and ascites are often described, hepatic encephalopathy is much rarer, with only a few case reports, often related to an advanced and untreated disease (27–29). To our knowledge, there is only one study specifically looking on hepatic sarcoidosis with PH (symptomatic or not), conducted in 1987, describing seven patients, associated with a report of 40 additional cases from the literature, among which 32 had symptomatic PH (10). In this study, the authors distinguish three different groups of patients: a first group with often symptomatic PH complicating chronic intrahepatic cholestasis with severe cirrhosis and signs of hepatocellular insufficiency, a second with PH (mainly symptomatic) without prominent cholestatic features, and a third group (of three patients) with chronic intrahepatic cholestasis with early prominent PH but moderate portal fibrosis.

We conducted a literature review between January 1988, after Valla et al.’s review (10), and December 2021, bringing together every article related to hepatic sarcoidosis with symptomatic PH. A total of 15 additional articles and 17 patients were found, mainly as case reports, often with missing data, especially on patient’s follow up. The description of these articles is summarized in Table 3. Some retrospective studies mentioning patients with symptomatic PH were not included due to lack of data on these patients and their evolution (19, 30).

**TABLE 3:** Hepatic sarcoidosis complicated by symptomatic portal hypertension: Summary of literature search.

References	Patient	Hepatic comorbidities	Hepatic symptoms	LFT at diagnosis	Cirrhosis at diagnosis	Extrahepatic disease	Treatment	Evolution
Melissant et al. (20)	58, M Caucasian		Variceal bleeding	Cytolysis cholestasis	No	MP, heart	Steroids	Favorable 1 year
Moreno-Merlo et al. (32)	52, M African		Ascites variceal bleeding	Cytolysis cholestasis	No	MP	Sclerotherapy	Death infection portal vein thrombosis
	53, M African		Ascites variceal bleeding encephalopathy	Normal	No	MP	Sclerotherapy liver transplantation	Portal vein thrombosis
Wong et al. (37)	27, M		Variceal bleeding	Cholestasis	No	MP	Sclerotherapy	Death HCC
Gavillan (50)	33, F		Ascites	Normal	Yes	0	Liver transplantation	Favorable 21 months
Cengiz et al. (47)	43, F Africo-Caribbean	Diabetes	Ascites encephalopathy	Cytolysis cholestasis	Yes	Eye, PNS	TIPS steroids liver transplantation	Recurrent hepatic sarcoidosis
Vannozzi et al. (26)	25, F		Ascites variceal bleeding	Cholestasis	No	Mesenteric lymphadenopathy	Steroids sclerotherapy	Favorable 10 years
Mosea et al. (51)	75, M		Variceal bleeding	Normal	Yes	MP	Steroids	Favorable 5 years
Yoshiji et al. (23)	46, F		Ascites variceal bleeding	Cytolysis cholestasis	Yes	MP	Missing data	Missing data
Tan et al. (11)	48, M African	Hypertension	Ascites	Cytolysis cholestasis	Yes	MP	Steroids	Considering liver transplantation
Ivonye et al. (52)	41, M African	Diabetes	Ascites variceal bleeding	Normal	No	MP	Diuretics	Missing data
Van Brusselen et al. (53)	7, M Caucasian	Liver transplantation	Ascites Budd-Chiari	Cytolysis cholestasis	No	0	Steroids	Favorable
Saito et al. (22)	46, F		Ascites variceal bleeding	Cholestasis	Yes	MP, heart, skin,	Steroids	Favorable
Achakzai et al. (21)	13, M		Ascites	Normal	Yes	0	Steroids	Favorable
Sedki et al. (12)	56, F African	Metabolic syndrome	Encephalopathy	Cytolysis cholestasis	Yes	MP, skin	Liver transplantation	Favorable
	65, M Caucasian		Ascites	Unknown	Yes	0	Steroids, MTX, liver transplantation	Acute graft rejection
Moretti et al. (54)	62, F	Diabetes	Variceal bleeding	Normal	Yes	MP	Steroids	Missing data

F, female; M, male; MP, mediastino-pulmonary; PNS, peripheric nervous system; MTX, methotrexate; TIPS, transjugular intrahepatic porto-systemic shunt; HCC, hepatocellular carcinoma.

There was a slight male predominance (59%), and despite a lack of data, African origin seemed to be more important, consistent with the higher prevalence in this population (4). As in our cohort, there was a majority of patients with impaired liver function tests at diagnosis. Extrahepatic disease was mainly represented by mediastino-pulmonary involvement (11/17), then skin and cardiac damage (2/17 each). Four patients only had liver involvement. Ascites was present in 12 patients, while variceal bleeding in 10. Encephalopathy was much rarer (4/17), consistently with our cohort. Budd-Chiari syndrome was found in one patient, as portal vein thrombosis was present in two, but complicating already advanced PH.

PH was associated with confirmed cirrhosis stage on liver biopsy in 10 patients (59%), confirming the many pathways that can lead to PH in hepatic sarcoidosis. Evolution seemed to be surprisingly favorable in almost half of cases, but often with a short follow-up period. Again, information on therapeutic care, from symptomatic to etiological treatments, as well as its efficacy and tolerance, were often missing.

This review of the literature, associated with our report of 12 patients, is the second after Valla’s in 1987 to focus specifically on PH in hepatic sarcoidosis. It underlines the high mortality of hepatic sarcoidosis, once the stage of symptomatic PH is reached, and should encourage early therapeutic intensification followed by increased surveillance, in patients not necessarily having cirrhosis.

Indeed, the pathogenesis of PH in sarcoidosis is not clear and may involve multiple mechanisms (10, 11). It has been suggested that small arterio-venous shunts may form in the region of the granulomas, resulting in an elevated portal blood flow which subsequently increases intrahepatic resistance (11). In the absence of cirrhosis, presinusoidal block due to portal granulomas or compression of the portal vein by involved portal lymph nodes could induce PH (10, 11, 31). Some authors have also hypothesized the role of a vascular involvement by granulomatous phlebitis of the portal and hepatic veins in hepatic parenchyma (32). As well, Budd–Chiari syndrome or portal vein thrombosis can also be encountered in hepatic sarcoidosis, although their presence seems exceptional (33–35). Because of the small size of most studies, the prevalence of cirrhosis related PH varies a lot, from 27 to 100% (5, 9, 12, 36). Half of our patients with hepatic sarcoidosis already had cirrhosis at the time of diagnosis and only four patients (33.3%) developed PH without cirrhosis. No Budd-Chiari syndrome was found in this cohort, and its description in hepatic sarcoidosis remains limited to case reports (33, 34).

Hepatocellular carcinoma complicating hepatic sarcoidosis seems to be exceptional with less than 5 case reports in the literature (18, 37) and no patient in our cohort.

The treatment of hepatic sarcoidosis is not codified, as there are no randomized controlled trials. It is so difficult to draw conclusions on the efficacy and long-term benefits of glucocorticoids and other immunosuppressive therapies, and the response to conventional immunosuppression is variable and unpredictable (13). Corticosteroids are considered as the mainstay of therapy. In second line treatment, antimetabolites such as azathioprine and methotrexate are the most common agents used (38). As shown in the hepatic sarcoidosis literature, we observed that corticosteroids are often ineffective in first-line treatment of hepatic sarcoidosis and require a second line treatment (5, 19). It may also improve liver test without improvement in liver histology, namely granulomatous inflammation (10, 12, 39). Such as for corticosteroids, the efficacy of second-line treatments has not been specifically evaluated for hepatic sarcoidosis. This is likely due to the rarity of symptomatic hepatic sarcoidosis without other extrahepatic manifestations, with mostly case reports, retrospective cohort or systemic sarcoidosis studies (10).

The main purpose of treatment is to control symptoms and finally prevent progression to cirrhosis and/or PH. Reached this stage, the management of patients can be similar to that of other causes of cirrhosis and/or PH, including medical treatment such as β-blockers, sclerotherapy of esophageal variceal, or TIPS (40). Yao et al. studied the efficacity of TIPS for non-cirrhotic PH over 25 patients, including three patients with sarcoidosis (41). The indication of TIPS was esophageal varices for two patients and ascites for the other one. Among these three patients, all of them experienced early or late encephalopathy and two patients died during the median follow-up of 39 months (41). Two patients of our cohort benefited from TIPS, also with a poor result (postoperative death and recurrence of ascites).

In patients with sarcoidosis, once liver involvement has been confirmed, it seems important to screen for signs of PH, especially screening for esophageal varices. To our knowledge, the 2015 Baveno VI guidelines against performing upper gastrointestinal endoscopy in patients with compensated cirrhosis who have a liver stiffness < 20 kPa and a platelet count > 150,000/mm^3^ was not evaluated in this population, but would be of interest (42). Then, the management and follow-up of patients could be modeled on the recommendations of other causes of PH, by looking in particular for the predictive factors of digestive bleeding (43, 44).

In this series, it is difficult to assess the efficiency of the treatments, as at the diagnosis of hepatic sarcoidosis, half of patients already presented cirrhosis and a third signs of PH. On the other hand, five patients presented serious infectious complications, including two deaths. Introduction of an immunosuppressive agent should be well thought out, in patients who may already be immunosuppressed in case of associated cirrhosis. Furthermore, hepatic comorbidities were present in seven of our 12 patients and in three of five patients who presented signs of hepatocellular insufficiency. Screening and management of hepatic comorbidities seems so essential.

Finally, in cases of end-stage liver disease or refractory PH, orthotopic liver transplantation may be proposed (45). Currently, only 0.01% of liver transplantations in the United States are attributed to end-stage liver disease related to hepatic sarcoidosis, with good post transplantation outcomes and survival rates similar to other liver diseases (46). Recurrence of sarcoidosis in the transplanted liver can occur in about 30% of patients, and is generally treated with corticosteroids to prevent damage and complications in the allograft (46, 47). Disease recurrence has not been shown to cause allograft dysfunction (46). In our cohort only two patients received liver transplantation with a good result, without recurrence of the sarcoidosis thereafter. In case of hepatic sarcoidosis with symptomatic PH, liver transplantation should be quickly considered when corticosteroids fail, given the low efficacy of specific management of PH, but also of immunosuppressive second-line treatments and their infectious risk.

Mortality from hepatic sarcoidosis may represent up to 5% of all causes of death in patients with chronic sarcoidosis (14). Ungprasert et al. also showed that liver involvement was a significant prognostic factor for death among patients with sarcoidosis, with hazard ratio of 5.79, after adjustment for age, sex and calendar year of sarcoidosis diagnosis (5). More recently, Graf et al. showed increased mortality when associated with end-stage liver disease (19). In our cohort of severe hepatic sarcoidosis, more than half of patients (58.3%) died, including 4 patients on digestive hemorrhages, supporting this idea. There are very few data on the mortality of symptomatic PH in sarcoidosis: Valla et al. reported that gastro-intestinal bleeding was the cause of death in three of seven patients who had a bleeding event (10). However, mortality related to hepatic sarcoidosis appears to be much lower compared to other liver diseases, such as non-alcoholic or alcoholic liver cirrhosis, which can be more than 50% at 5 years (5, 48, 49).

This study has several limitations, the main ones being related to its retrospective design and the small number of patients. Moreover, some of the pertinent data were not available. Imaging study and biochemical tests were obtained at physician discretion which could introduce surveillance bias. As a majority of patients were of Caucasian origin, generalizability of the observations to other populations may be limited. In addition, more than half of patients had hepatic comorbidities, such as alcoholic hepatitis or non-alcoholic steatohepatitis, it is therefore difficult to interpret the precise impact of sarcoidosis in liver damages, and the evolution of these patients could be marked as much by the sarcoidosis as by these comorbidities. Finally, all patients came from tertiary hospitals, which may result in a different pattern of hepatic sarcoidosis complications detection and management.

As previously explained, the pathogenesis of hepatic sarcoidosis is unclear and may involve several mechanisms. It might be interesting to look for anatomopathological findings on liver biopsies and compare them with the patient’s evolution, especially to see if there is a predictive pattern toward PH.

The high mortality of patients with symptomatic PH should prompt prospective studies to determine the best management in patients who have not reached this stage.

## Conclusion

This study highlights the high morbidity and mortality of hepatic sarcoidosis with symptomatic PH, including the risk of digestive bleeding in the presence of esophageal varices, but also serious infections, with or without a cirrhotic stage. Tolerance profile and efficacy of the treatments appear to be poor, whether it is the specific treatment of sarcoidosis or the management of PH. Then, liver transplantation should be quickly considered when corticosteroids fail.

## Data availability statement

The raw data supporting the conclusions of this article will be made available by the authors, without undue reservation.

## Ethics statement

The studies involving human participants were reviewed and approved by the HCL Institutional Review Boards (Comité d’Ethique du CHU de Lyon) (N°20-116). Written informed consent for participation was not required for this study in accordance with the national legislation and the institutional requirements.

## Author contributions

MF and PS contributed equally to the design and implementation of the research, data collection, analysis of the results, writing of the manuscript, and took responsibility for the integrity and accuracy of the data. GR and AD-B contributed to the data collection. MF, GR, AD-B, ML, MG-V, ID, YJ, FB, MM, and PS revised the manuscript. All authors read and approved the final version of the manuscript.
